# Self-reported sleep quality, weight status and depression in young adult twins and siblings

**DOI:** 10.1186/s40608-015-0079-8

**Published:** 2015-12-23

**Authors:** Alexia Sawyer, Abi Fisher, Clare Llewellyn, Alice M. Gregory

**Affiliations:** Health Behaviour Research Centre, Department of Epidemiology and Public Health, University College London, Gower Street, London, WC1E 6BT UK; Department of Psychology, Goldsmiths, University of London, New Cross, London, SE14 6NW UK

**Keywords:** Sleep, Obesity, Overweight, Depression, Young adults

## Abstract

**Background:**

Research supporting relationships between sleep quality, weight, depression and anxiety has typically examined the relationships separately rather than simultaneously, potentially hampering insights into the characteristics of reported links. This study aimed to fill this gap in the research to provide further insight into the factors associated with sleep.

**Methods:**

Data from wave 4 of the G1219 cohort were used in cross-sectional analyses. The sample comprised 1392 adult twins and siblings aged 18–27 years. Participants completed a self-report questionnaire which included the Pittsburgh Sleep Quality Index as a measure of sleep quality, the Short Mood and Feelings Questionnaire as a measure of depression symptoms and the Revised Symptoms of Anxiety Scale as a measure of anxiety symptoms. Participants were asked to self-report general health and weight and height so researchers could derive weight status from measures of body mass index.

**Results:**

An analysis of covariance including weight status, depression, anxiety and general health as predictors and sleep quality as the outcome revealed main effects of depression (F(3,1163) = 10.93, *p* < 0.001) and general health (F(4,1163) = 5.72, *p* < 0.001) only.

**Conclusions:**

A direct relationship between weight and sleep should not be assumed as it is possible that the relationship is at least in part accounted for by depression symptoms or general health. Depression symptoms and general health may also account for the association between sleep quality and anxiety symptoms in young adults.

## Background

As the importance of sleep to physical and psychological health becomes increasingly clear, there is a growing need to establish the mechanisms through which the relationships operate [1–3]. Understanding such characteristics of the relationships will aid in determining the direction of effects and thereby identify opportunities for intervention to improve health outcomes [4].

One of the most robust relationships reported within the sleep-health literature is that between sleep quality and symptoms of anxiety and depression [5–9]. Indeed, disturbed sleep is listed within the DSM IV and V as one of the principle symptoms of some clinical anxiety and depressive disorders [10, 11]. A link is also supported in epidemiological research, where community-dwelling individuals with disturbed sleep are more likely to present symptoms of depression and anxiety [8]. Recent studies exploring the aetiology of this relationship using data from the current study and, elsewhere, a youth twin sample, found genetic factors of the three variables to be moderately correlated – supporting the conceptualisation of sleep quality as a symptom of depressed or anxious moods but also warranting independent consideration and investigation into the direction of relationships [7, 12].

Emerging evidence also supports a relationship between poor quality sleep and overweight and obesity in adulthood. Building on an evidence-base documenting an established link between sleep duration and weight status (e.g. [3, 13–15]), Jennings et al. [16] reported an association between self-reported sleep quality and a number of indicators of metabolic syndrome including body mass index (BMI) and waist circumference. In addition, in a sample of 53 healthy adults, self-reported sleep quality was related to eating behaviours which promote overweight and obesity such as hunger and more disinhibited, uncontrolled and, notably, emotional eating [17]. Interestingly, while depression was not significantly associated with the weight-related outcomes, controlling for depression did attenuate the relationship between sleep quality and some weight-related outcomes (presence of metabolic syndrome and hunger) in both studies, suggesting a role for depression in the sleep quality-weight relationship. However, these studies were limited by comparatively small sample sizes and no consideration of other aspects of psychological health such as anxiety symptoms. Moreover, the direction of causality from sleep characteristics to weight status cannot be assumed (for example, in a study revealing cross-sectional relationships between objective sleep duration and BMI, a predictive relationship was not revealed [18]). Therefore, there is a need in the literature to examine sleep both as an exposure and an outcome.

In order to advance current understanding of the pathways and mechanisms linking sleep to weight, depression and anxiety, it may be valuable to elucidate the broader interplay between the variables. Exploring factors simultaneously rather than separately could prove advantageous in ensuring closely related variables that could confound or act as causal intermediates are adequately considered. In fact, it has been previously proposed that inconsistent adjustment of psychological factors such as depressive or anxiety symptomatology in the relationship between weight and sleep might have led to inconsistent findings in the literature [17, 19]. Similarly, adjustment for general physical health could help to remove confounding noise in the reported relationships [19].

This study used a young adult sample from the G1219 cohort to investigate the relationship between sleep quality, weight, depression and anxiety. Previously, symptoms of depression and anxiety have been found to be moderately correlated with sleep disturbance in this sample [7]; however the possible role of symptoms of depression and anxiety in a relationship between weight and sleep quality has not yet been investigated.

## Methods

### Participants

This study used data from wave 4 of the G1219 and G1219 Twins longitudinal study (the only wave at which sleep had been assessed at the time of analysis). The G1219 cohort comprises a random selection of twins born in the United Kingdom (UK) between 1985–1988 and a sample of sibling pairs originating from the GENESiS study (for details see [20–22]). A total of 3640 individuals aged 12–19 years participated in the first wave of data collection. Individuals participating in waves 2 and 3 of data collection were traced for wave 4 data collection. Of the 2550 individuals who were successfully traced, 1556 individuals were included in the wave 4 dataset (61 % of those traced at wave 4; 74 % of those participating in wave 3). A total of 1422 participants had complete height and weight data. Those who were severely underweight (BMI of <16.00; *n* = 21) or severely obese (BMI of ≥40.00; *n* = 9) were excluded from the sample as participants of extreme weight (classified as such in World Health Organisation weight tables [23]) could influence the findings of the study. There was also one outlying participant with extremely poor sleep quality (score = 18.67) who was excluded from analyses. This left a sample of 1392 for the current study. The G1219 cohort at wave 1 differed slightly from the general UK population in that levels of parental education were somewhat higher in the cohort (39 % educated to A-level or above) and G1219 parents were more likely to own their home (82 % owned their own home [21]). There were also slight differences in the demographics of the participants lost to attrition between wave 1 and 4, as might be expected with large cohort studies.

Ethical approval for different stages of the G1219 studies was granted by the Research Ethics Committees of the Institute of Psychiatry, South London and Maudsley NHS Trust, and Goldsmiths, University of London. Informed written consent was obtained from participants.

### Measures

#### Sleep quality

Sleep quality over the previous month was self-reported using the Pittsburgh Sleep Quality Index (PSQI [24]). This 19-item questionnaire produces a global sleep quality score with possible values from 0 (no difficulty sleeping) to 21 (severe difficulty sleeping). The PSQI corresponds well to other measures of sleep such as sleep diaries [24] and demonstrates good internal consistency and test-retest reliability in the 0.8 range [25]. The PSQI had a Cronbach’s alpha of 0.71 in our sample [7].

#### Weight status

Self-reported measures of weight and height were used to compute BMI (kilograms/meters^2^). BMI was categorised using standard cut-offs: underweight (BMI = 16–18.5), healthy weight (BMI = 18.5–24.9), overweight (BMI = 25–29.9) and obese (BMI 30–39.9).

#### Depression and anxiety symptoms

Depression symptomatology over the previous 2 weeks was measured using the Short Mood and Feelings Questionnaire (SMFQ [26]). This questionnaire includes 13 items such as “I cried a lot” and “I did everything wrong” which are each scored on a 3 point Likert scale (0 = ‘not true’; 2 = ‘true’) and summed for a total score tapping key symptoms of depression. The SMFQ has been shown to have satisfactory internal reliability elsewhere [26–28] and good internal consistency within this dataset (Cronbach’s alpha = 0.90 in the whole sample [7]). The SMFQ does not include a direct measure of sleep quality or weight status.

Anxiety symptoms were measured using the Revised Symptoms of Anxiety Scale [7] – a 36-item questionnaire (responses scored on a 4-point Likert scale: 0 = ‘never’; 3 = ‘always’) that measures DSM IV symptoms of 5 anxiety subtypes (social anxiety, separation anxiety, panic/agoraphobia, obsessions/compulsions and general anxiety). A summed score was used in analyses as the subtypes have been shown to co-occur [29] and have a similar magnitude of association with sleep disturbance within this sample [7]. This questionnaire is an adapted version of the Revised Children’s Anxiety and Depression Scale which can be used with adults and has excellent internal reliability (Cronbach’s alpha = 0.94 [7, 30]). No items directly measure weight status and although three items tap sleep, previous analyses using this dataset demonstrated that excluding these items from the scale did not substantively impact the reported relationship between sleep quality and anxiety [7]. It was therefore considered appropriate to include the full scale in current analyses.

#### General health

Participants self-reported their health as “excellent”, “very good”, “good”, “fair” or “poor” using a single item.

#### Covariates

Socio-demographic factors thought to be related to sleep, weight status and/or depression and anxiety (sex, age, level of highest education qualification [education], status as an ex-, current or non-smoker [smoker status] and frequency of alcoholic intake occasions [alcohol intake]) were self-reported [8, 19].

### Statistical analyses

Univariate analyses of variance (ANOVAs) investigated independent effects of weight status, depression, anxiety and general health on sleep quality, using the full sample. An analysis of covariance (ANCOVA) including weight status, depression, anxiety and general health as predictor variables and sleep quality as the outcome was also conducted. Socio-demographic factors significantly related to weight status (age, sex, smoker status, education and alcohol intake) were included in a final multivariate model as covariates.

In order to strengthen inferences about a possible relationship between weight and sleep, sibling-comparison design analyses were performed [4]. This design compares siblings on a trait (e.g. sleep quality) and looks to see whether the sibling with the higher score on that trait is also the sibling scoring more highly on another trait (e.g. weight). This process effectively removes the impact of shared environment on associations, hence showing that associations are not simply a function of an aspect of the environment making family members more alike on certain traits (e.g. low socio-economic status (SES) leading to poorer sleep and increased weight). This is a discordance design which can be used within studies of siblings to control for shared familial confounders (such as environment) that may otherwise remain unmeasured. As such, they can make a useful contribution to a wider body of evidence aiming to establish the association and build up a picture of whether one variable may cause another [31]. Sibling differences in BMI were calculated by subtracting the BMI of one sibling from the other and allocating each sibling within pairs discordant for BMI to either a ‘higher’ or ‘lower’ BMI group. Not all siblings were discordant for BMI; when they were discordant, only one BMI discordance score for each pair was included in analyses, giving a sample of *n* = 380. An ANOVA and ANCOVA were conducted to explore the effect of sibling differences in BMI on sleep quality (i.e. whether the sibling of greater weight was more likely to also have poorer sleep quality and vice versa) and then adjusting for individual scores of depression, anxiety and general health. These analyses tested whether siblings with a greater BMI were more likely to experience poor sleep quality than their lighter siblings after controlling for some of the unmeasured noise arising from a shared familial environment. All analyses were carried out with a randomly selected individual within each family pair in SPSS v 20. Statistical significance was set at *p* < 0.05.

## Results

### Descriptive statistics

Participants were aged between 18 and 27 years (mean 20.3 years) and 71.8 % were of a healthy weight (mean BMI = 22.58; range = 16.09 – 38.79). Participant characteristics are displayed in Table 1. Depression and anxiety scores were positively skewed - as may be expected with measures of emotional difficulties, so were categorised into quartiles (depression: skew = 1.26 [SE = .062]; anxiety: skew = 1.18 [SE = .062]). Sleep quality was not skewed so was treated as continuous (skew = 0.89 [SE = .063]).

**Table 1:** Participant characteristics for study sample (mean and SD unless otherwise stated)

	N (%)	Mean (SD)
Age (years)	-	20.36 (1.78)
Sex (n;%)		
Male	528 (38)	-
Female	864 (62)	-
Education		
Up to GCSE or GNVQ	225 (16.3)	-
AS-level or A-level	702 (50.9)	-
Above A-level	453 (32.8)	-
Smoking		
Yes	278 (20)	-
Given up	135 (10)	-
Never	977 (70)	-
Alcohol intake		
Up to twice a month	436 (32.9)	-
Once or twice a week	621 (46.8)	-
More than twice a week	269 (20.3)	-
Weight status		
Underweight (BMI: 16–18.5)	123 (8.8)	-
Healthy weight (BMI: 18.5–29.9)	1000 (71.8)	-
Overweight (BMI: 25–29.9)	200 (14.4)	-
Obese (BMI: 30–39.9)	69 (5.0)	-
Sleep quality^a*^	-	5.71 (3.01)
Depression^b*^	-	6.43 (5.71)
Anixety^c*^	-	25.05 (14.74)
General health^d*^	-	2.33 (0.97)

^a^Sleep quality was assessed using the sum score from the Pittsburgh Sleep Quality Index. [38] ^b^Depression symptoms were measured using the Short Moods and Feelings Questionnaire.[26] ^c^Anxiety symptoms were measured using the Revised Symptoms of Anxiety Scale.[7] ^d^General health was measured using a single, self-report item. *Higher scores for sleep quality, depression, anxiety and general health indicate poorer sleep quality, greater number of depression symptoms, greater number of anxiety symptoms and poorer health, respectively

### Relationship between sleep quality, weight status, anxiety and depression

A trend of an effect of weight status (F(3,1361) = 2.50, p = 0.058) and significant effects of depression symptoms (F(3,1512) = 142.47, *p* < 0.001), anxiety symptoms (F(3,1514) = 76.20, *p* < 0.001) and general health (F(4,1509) = 49.69, *p* < 0.001) on sleep quality were reported in univariate models (Table 2). A linear, dose–response relationship between depression, anxiety and general health and sleep quality was reported, whereby individuals reporting more depression symptoms or poorer general health experienced significantly poorer sleep quality and a U-shaped relationship between weight status and sleep quality was revealed in weight analyses (Table 2). In a multivariate model including weight status, depression, anxiety and general health, only depression (F(3,1163) = 10.93, *p* < 0.001) and general health (F(4,1163) = 5.72, *p* < 0.001) significantly predicted sleep quality (Table 2). No interactions were significant. As shown in Table 2, main effects of depression (F(3,1085) = 10.33, *p* < 0.001) and general health (F(4,1085) = 5.25, *p* < 0.001) remained when controlling for covariates, with sex (F(1,1085) = 6.43, p = 0.011) being the only significant covariate of the relationship.

**Table 2:** ANOVA and ANCOVA results with sleep quality as the outcome

	N	Sleep quality^a*^ Mean (SE)	ANOVA *p*-value	ANCOVA Model 1^†^ *p*-value	ANCOVA Model 2^†^ *p*-value
Weight status			0.058	0.288	0.384
Underweight	116	6.27 (0.28)			
Healthy weight	984	5.58 (0.10)			
Overweight	199	5.92 (0.21)			
Obese	66	6.01 (0.37)			
Depression^b*^			<0.001	<0.001	<0.001
1 (fewer)	427	3.92 (0.13)			
2	416	5.26 (0.13)			
3	321	6.39 (0.15)			
4 (more)	352	7.69 (0.14)			
Anxiety^c*^			<0.001	0.368	0.204
1 (fewer)	428	4.42 (0.14)			
2	349	5.25 (0.15)			
3	368	5.93 (0.15)			
4 (more)	373	7.33 (0.15)			
General health^d*^			<0.001	<0.001	<0.001
1 (better)	298	4.34 (0.16)			
2	622	5.36 (0.11)			
3	419	6.23 (0.14)			
4	136	7.41 (0.24)			
5 (poorer)	39	9.01 (0.45)			

^†^Model 1 included weight status, depression, anxiety and general health; Model 2 included weight status, depression, anxiety and general health, adjusting for age, sex, smoking, education and alcohol. ^a^Sleep quality was assessed using the Pittsburgh Sleep Quality Index, with higher scores indicating poorer sleep quality.[38] ^b^Depression symptoms were measured using the Short Moods and Feelings Questionnaire.[26] ^c^Anxiety symptoms were measured using the Revised Symptoms of Anxiety Scale.[7] ^d^General health was measured using a single, self-report item. *Higher scores for sleep quality, depression, anxiety and general health indicate poorer sleep quality, greater number of depression symptoms, greater number of anxiety symptoms and poorer health, respectively

### Sibling-comparison analyses

An independent effect of sibling difference in BMI on sleep quality was reported (F(1378) = 6.58, p = 0.011), where siblings with a greater BMI reported poorer sleep quality. The effect of sibling difference in BMI on sleep quality was no longer significant after adjustment for depression, anxiety and general health (sibling difference in BMI: F(1279) = 0.159, p = 0.690) but there was a significant main effect of depression (F(3279) = 4.85, p = 0.003), anxiety (F(3279) = 3.53, p = 0.015) and a trending effect of general health (F(4279) = 2.34, p = 0.055), with individuals reporting more symptoms of depression, anxiety or poorer general health being more likely to experience poorer sleep quality.

## Discussion

Participants of a healthy weight or with fewer symptoms of depression or anxiety tended to report fewer sleep problems than their peers in this young adult sample. The relationship between weight status and sleep quality was U-shaped (i.e. healthy weight was associated with the best sleep – with those under and overweight/obese having poorer sleep) and was significant after controlling for a potentially confounding effect of shared familial factors such as shared environment. However, multivariate models suggested that a trend towards an association between sleep quality and weight status and a significant association between sleep quality and anxiety symptoms were not direct as associations became weaker when adjusting for depression symptoms. Depression, general health and sex were the only variables to remain significantly associated with sleep quality, with post-hoc analyses indicating a dose–response relationship between depression and general health and sleep quality.

This study contributes to previous research revealing a role for depression in the sleep quality-weight relationship and, for the first time to our knowledge, demonstrates it in a large young adult sample. Our findings also reflect the literature investigating a role for depression and general health in the link between weight and other components of sleep, in particular, sleep duration. For example, Calamaro et al. [32] reported that clinical depression played a role in the link between short sleep duration and increased weight in their sample of 13,568 adolescents. Moreover, Thomas et al. [33] found that physical health factors such as hypertension greatly attenuated the association between sleep duration and weight status, although, further adjustment for depression symptoms and emotional stress did not impact upon the explanatory power of the statistical model in this sample of adults (mean age 39.1 years). Interestingly, although depression was not a significant covariate of the relationship between sleep duration and weight in another sample of adults (mean age 45.3 years), physical health factors were [19].

It is important to note that the current study differs importantly from these studies. Firstly, sleep quality rather than sleep duration was the variable of interest. As it is possible that different aspects of sleep (e.g. length, quality) have different relationships with weight status, the majority of the literature views them independently; therefore the current study only looked at sleep quality. Secondly, our sample came from a young adult population. The relationship between weight and depression is less clear in older adults which may explain why physical health but not depression has been shown to confound the sleep duration-weight relationship in older populations [19, 33, 34]. Nonetheless, taken together these findings recommend that research examining the relationship between weight and sleep phenotypes (for example, quality, duration or chronotype) consider the potential role of depression and general health.

Although measures of sleep quality and depression symptomatology used in this study were self-reported and therefore open to recall bias and reduced accuracy in comparison with objective measures of sleep, the measures have good psychometric properties and have been validated against other measures of sleep, greatly strengthening our findings [24, 25]. The use of a non-clinical sample was also advantageous within the context of this study as it permitted exploration of the posited relationship between sleep and weight at a community level. However, it is important to note that our findings cannot be generalised to clinical samples and our sample differed slightly to the UK population in terms of parental education and home ownership, further limiting generalisability. Furthermore, the use of a self-reported, single-item measure of general health may constitute a weakness of the study; however, it has been shown elsewhere to have good psychometric properties and to correlate with physical functioning [34]. Additional limitations of the study may be the high number of participants of healthy weight, potentially obscuring true differences between groups and the use of self-reported measures of height and weight. The use of a young sample of relatively high SES may be partly responsible for the higher than expected number of healthy weight participants [35]. As with many other studies of this magnitude, objective measures of height and weight were not feasible, however it has been noted elsewhere that self-reports by young adults within community settings are satisfactory and can be used to accurately identify weight classifications [36, 37]. Nonetheless, future research would benefit from the additional inclusion of measurements of fat distribution such as body composition analysis or skinfold thickness. Further research may also aim to control other lifestyle information in analyses as factors such as physical activity expenditure and meal patterns may be important in the relationship between weight and sleep quality.

## Conclusions

This study supports a robust relationship between sleep quality and depression and suggests an important role for depression and general health in the putative link between weight status and sleep quality. Future research must ensure that these factors are appropriately controlled for in order to advance understanding of the sleep-weight relationship and properly manage current discussions around the regulation of weight through improvements in sleep quality.

## Abbreviations

ANCOVA: analysis of covariance
ANOVA: analysis of variance
BMI: body mass index
DSM: Diagnostic and Statistical Manual of Mental Disorders
PSQI: Pittsburgh Sleep Quality Index
SD: standard deviation
SE: standard error
SES: socio-economic status
SMFQ: Short Mood and Feelings Questionnaire
UK: United Kingdom
